# Supplementary material for: Use of a Molecular Switch Probe to Activate or Inhibit GIRK1 Heteromers In Silico Reveals a Novel Gating Mechanism
Source: Int J Mol Sci. 2022 Sep 16;23(18):10820. doi: 10.3390/ijms231810820 (PMC9502415; doi:10.3390/ijms231810820)
Supplement: Supplementary file 1 [file ijms-23-10820-s001.zip › ijms-1667510-supplementary.pdf]

International Journal of Molecular Sciences

MDPI

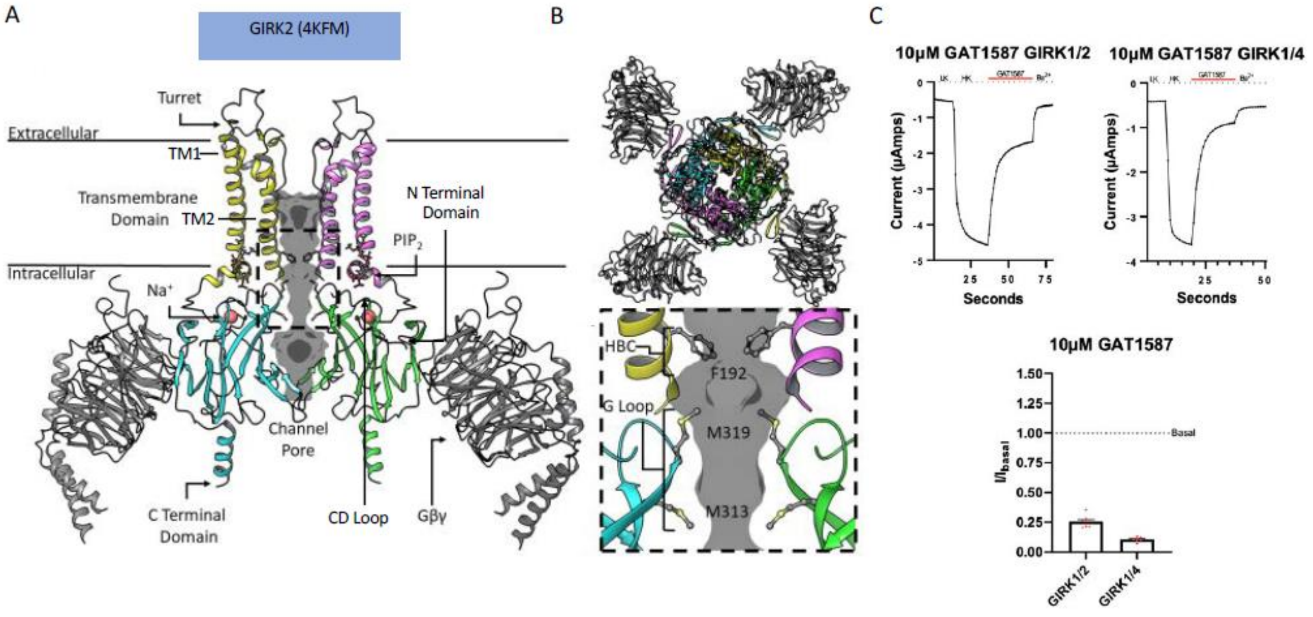

Figure S1. Pharmacological actions of a molecular switch moiety are reproduced in sub-microsecond SD simulations in Kir3.1/4. (**A**) Side view of GIRK2 homotetramers (4KFM) in a pre-open state in the presence of Gβγ, Na^{+}, and PIP_2. The two thick black lines indicate the position of the phospholipid bilayer. Bound Na^{+} (pink spheres) and PIP_2 (sticks) are shown. (**B**) The top-down view of the complex (top) and the two gates: the helix bundle crossing or HBC (F192) and the G loop (M313/M319). (**C**) K+ current time course responses from Xenopus oocytes expressing GIRK1/2 (top left) or GIRK1/4 (top right) heterotetramers seen when perfused with different solutions during TEVC recordings (see Material and Methods). Low K+: LK or ND96; High K+: HK or basal; Ba2+: 3 mM BaCl2. Responses to 10 M GAT1587 were normalized relative to the basal current (bottom).

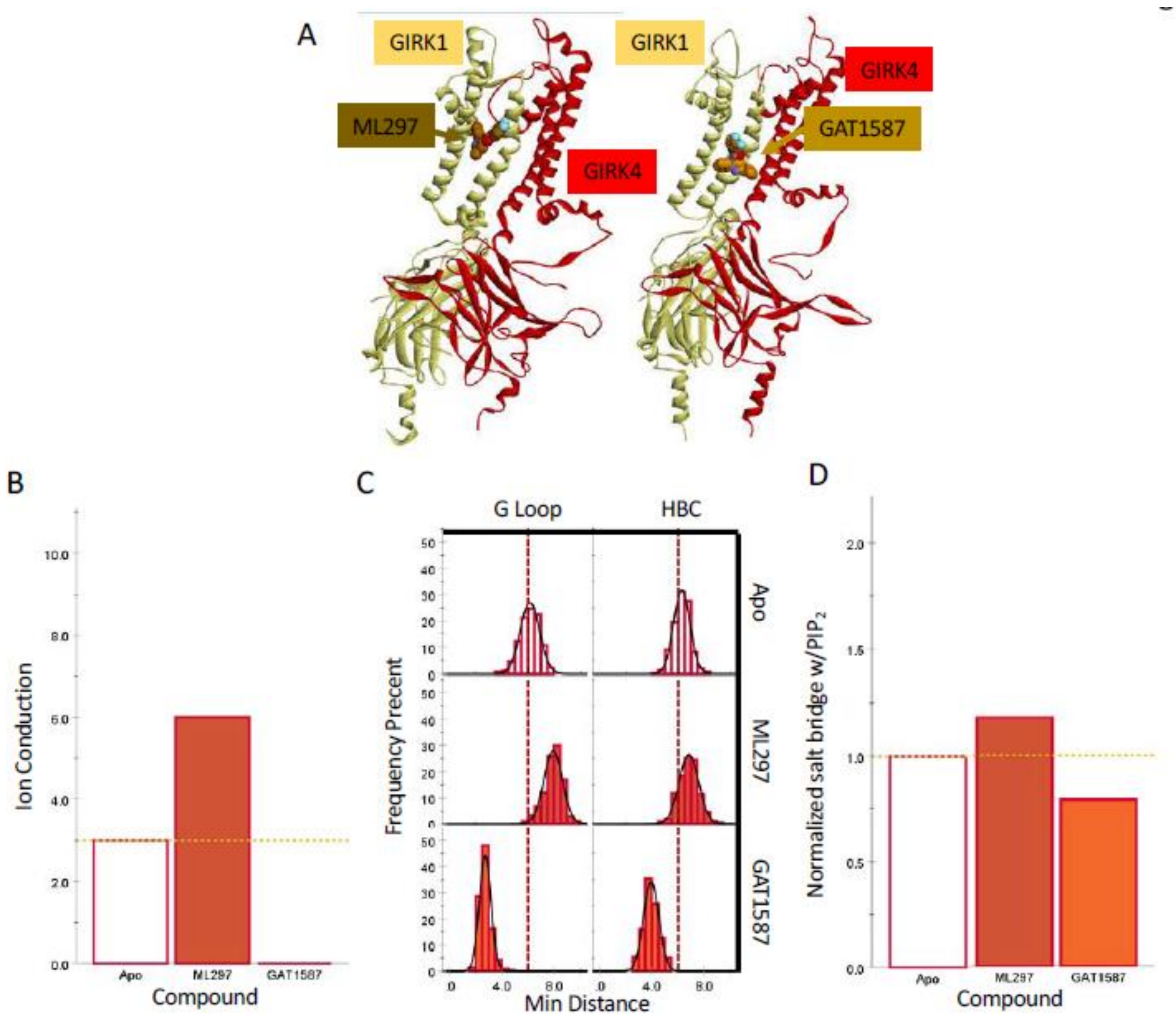

Figure S2. (**A**) Equilibrium binding site for ML297 and GAT1587 following a 300ns stochastic dynamics simulation. (**B**), (**C**), and (**D**) Ion conduction, distributions of minimum gate distances, and normalized salt bridge formation with PIP_2 over the last 150ns of the simulations from a single 300 ns MD run

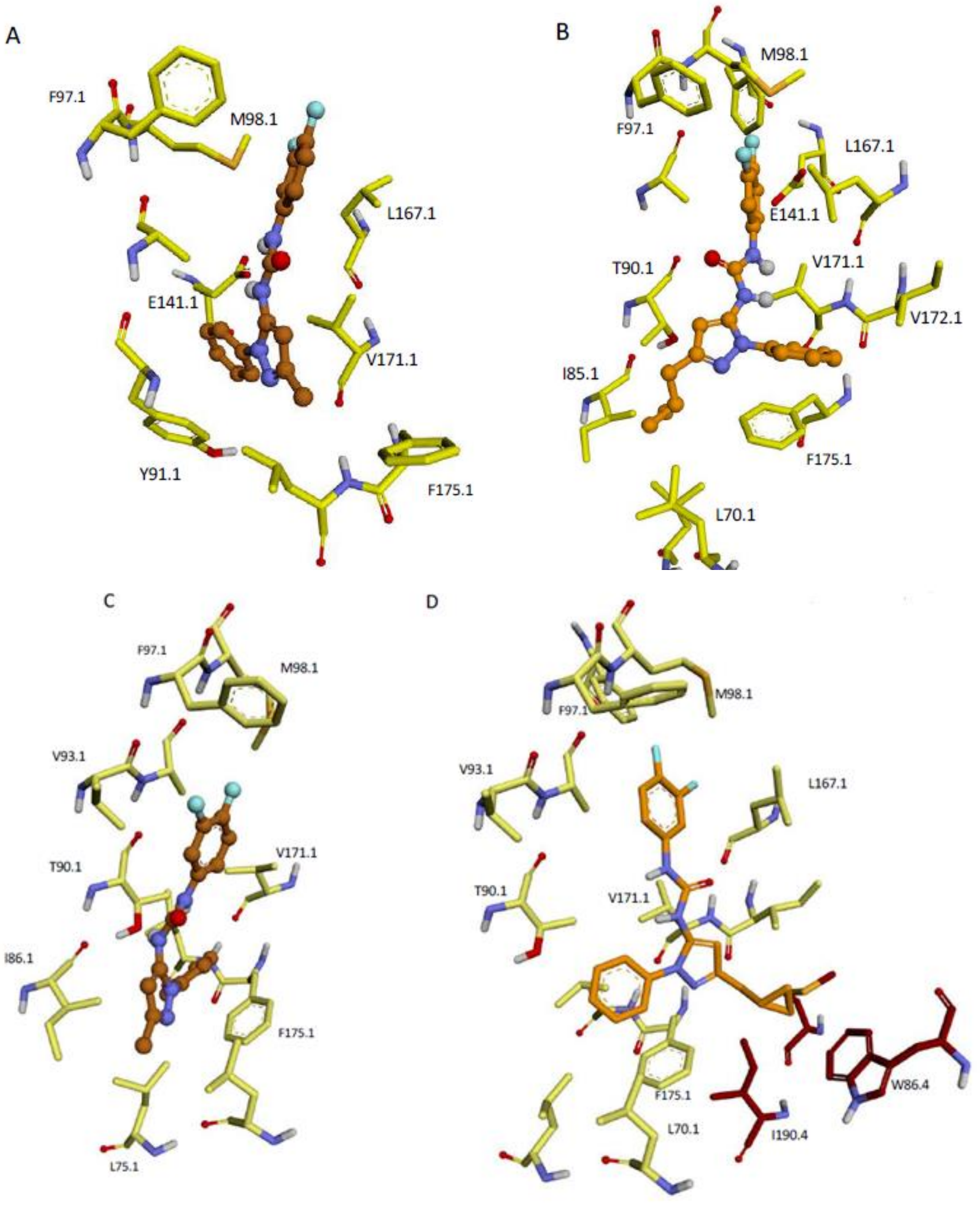

Figure S3. Docked poses of the activator (ML297) and inhibitor (GAT1587) to GIRK1/2 and GIRK1/4 heteromers. Docking poses of ML297(**A**) and GAT1587 (**B**) on the GIRK1/2 heterotetramer. Key residues in proximity to the ligands are highlighted. Docking poses of ML297(**C**) and GAT1587 (**D**) on the GIRK1/4 heterotetramer. Key residues in proximity to the ligands are highlighted. In contrast to the GIRK1/2 heteromer, GAT1587 docks proximally to some GIRK4 residues. Residues shown are within 4 angstroms from the compound binding site.

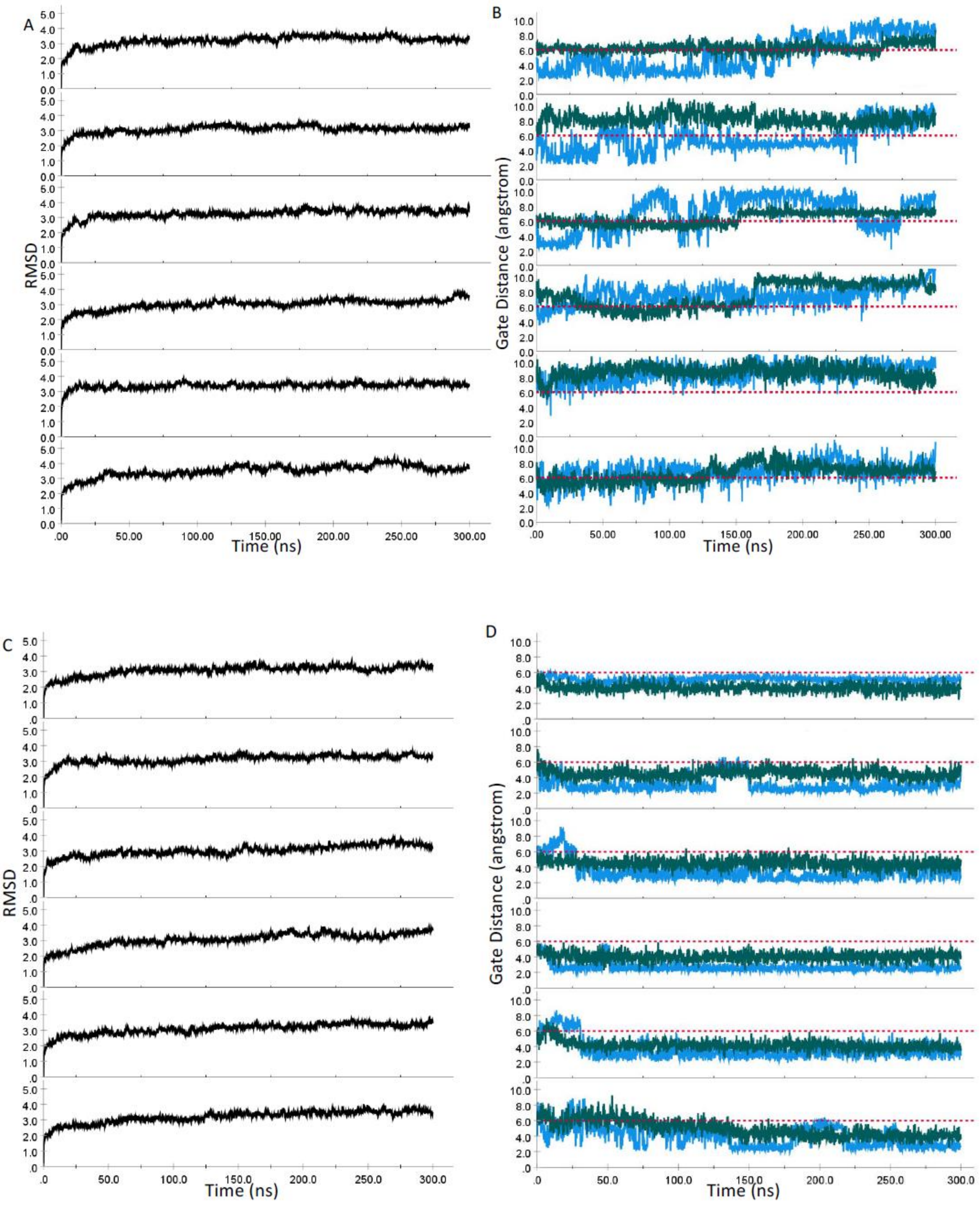

Figure S4. Replicas of 6-300 ns MD runs showing equilibration and behavior of the two gates of GIRK1/2. (**A**) The RMSD over the course of the simulation for each pose of ML297 on the GIRK1/2 is shown. (**B**) The channel gate

distances, the G-loop gate (blue) and the helix bundle crossing gate (green), are shown for the course of the simulation for each pose. (**C**) The RMSD over the course of the simulation for each pose of GAT1587 on the GIRK1/2 is shown. (**D**) The channel gate distances, the G loop gate (blue), and the helix bundle crossing gate (green), are shown for the course of the simulation for each pose.

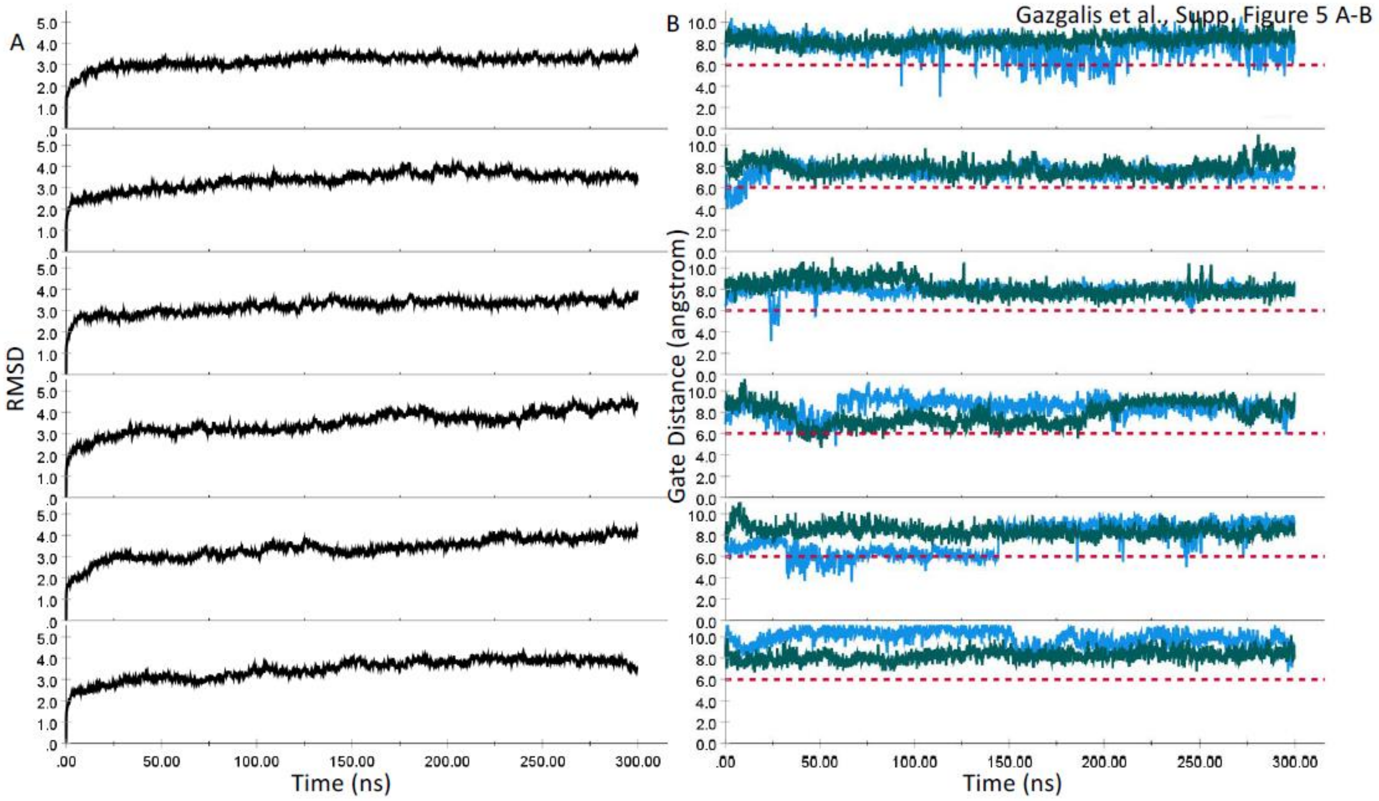

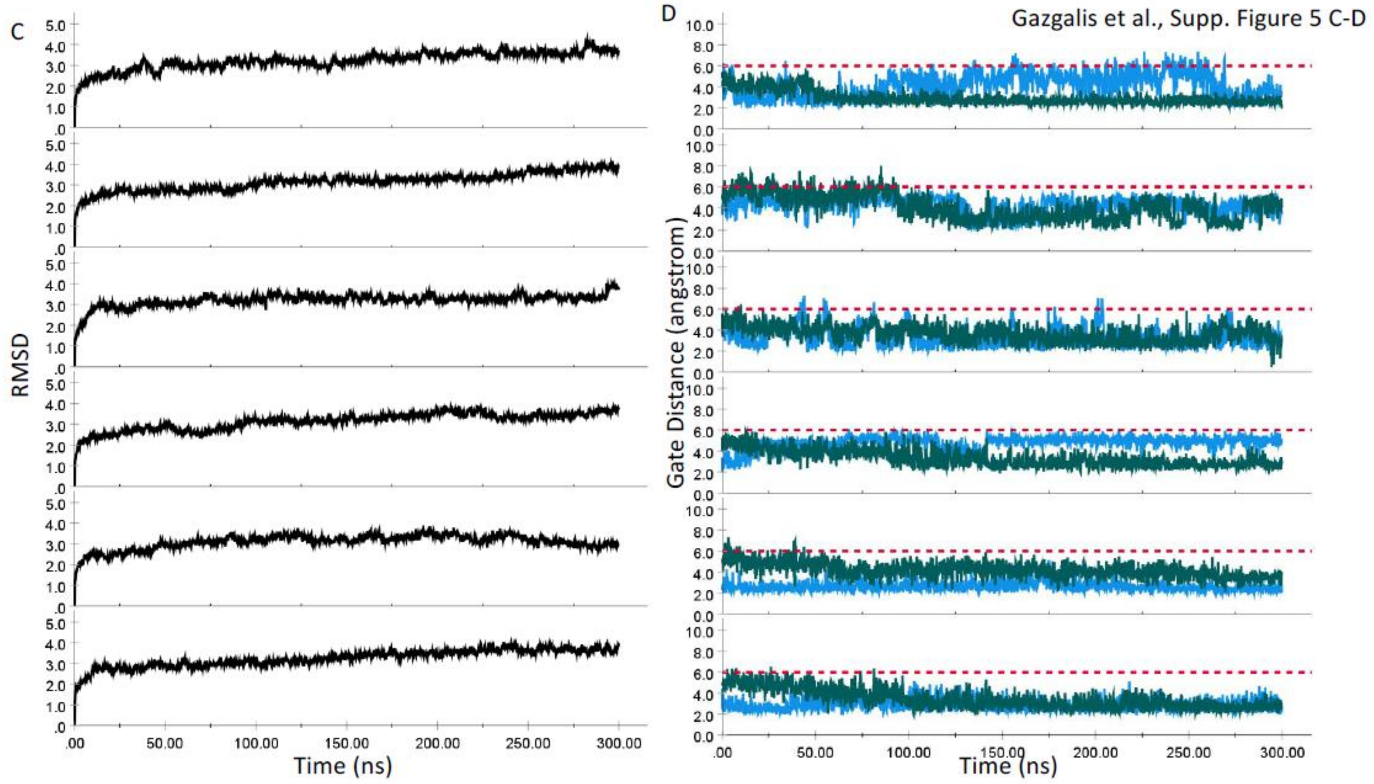

Figure S5. Replicas of 6-300 ns MD runs showing equilibration and behavior of the two gates of GIRK1/4. (**A**) The RMSD over the course of the simulation for each pose of ML297 on the GIRK1/4 is shown. (**B**) The channel gate distances, the G-loop gate (blue) and the helix bundles crossing gate (green), are shown for the course of the simulation for each pose. (**C**) The RMSD over the course of the simulation for each pose of GAT1587 on the GIRK1/4 is shown. (**D**) The channel gate distances, the G loop gate (blue) and the helix bundle crossing gate (green), are shown for the course of the simulation for each pose.

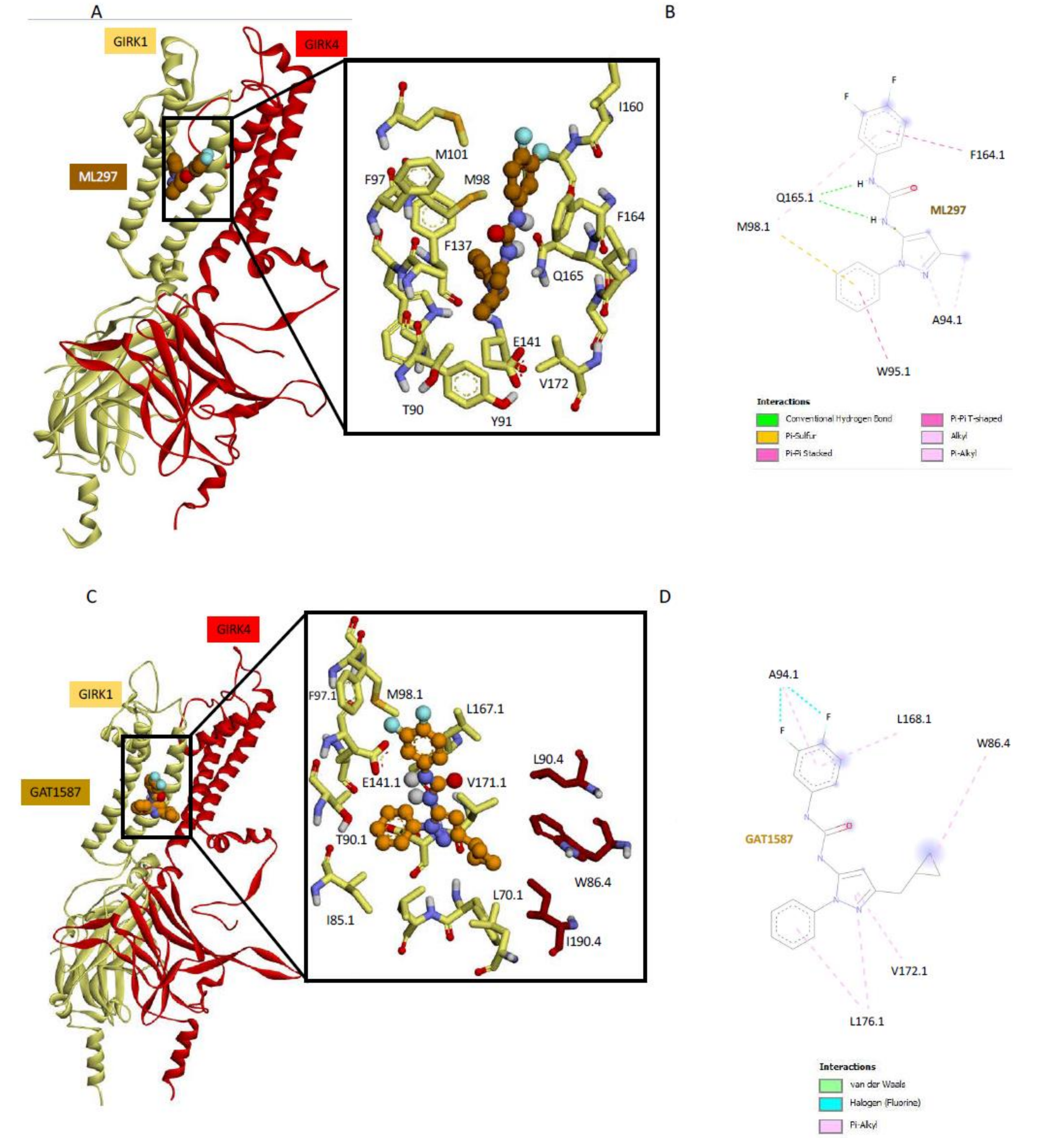

Figure S6. ML297 binding site between the TM1-TM2 helices of the GIRK1 subunit as part of the GIRK1/4 heterotetramer. (**A**) Equilibrium binding pose for ML297 when complexed with PIP_2 GIRK1/4 after 300ns of stochastic dynamics. (**B**) A 2D schematic representation of the compounds binding pose depicting protein ligand interactions and solvent accessibility. (**C**) Equilibrium binding pose for GAT1587 when complexed with PIP_2 GIRK1/4 after 300ns of

stochastic dynamics. (**D**) 2D schematic representation of the compound's binding pose depicting protein ligand interactions and solvent accessibility.

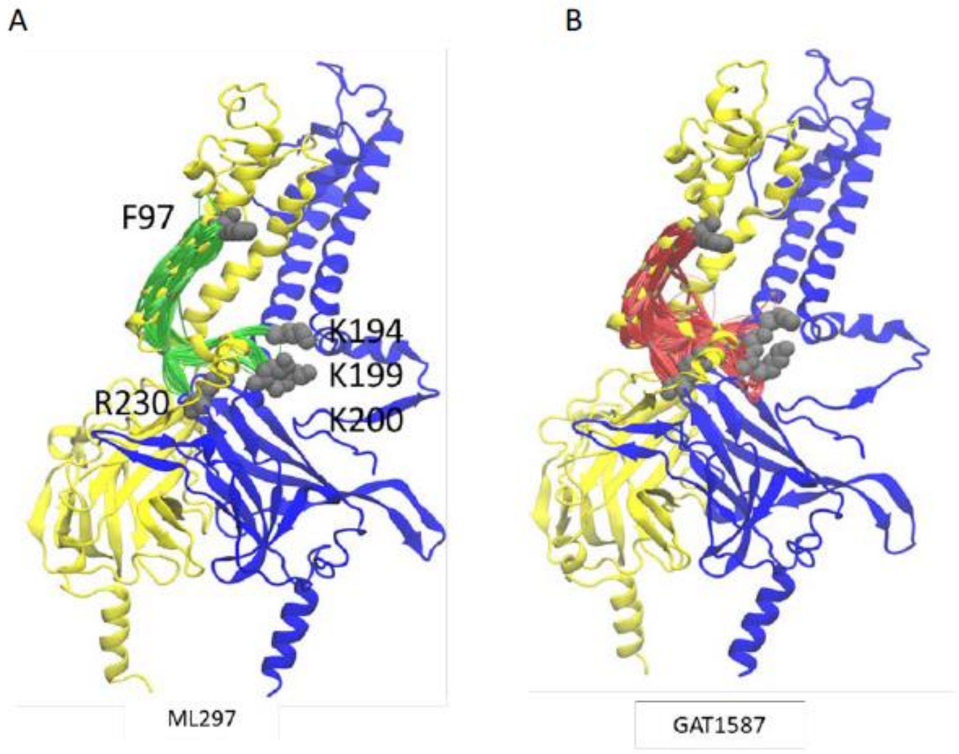

Figure S7. Shortest pathway analysis identifies correlated network of residues from ML297/GAT1587 bonding sites to key action sites of difference within the TM1 hydrophobic wire and the Slide Helix. (**A**) Results from the shortest pathway analysis of the ML297-mediated open state of GIRK1/2. (**B**) Results of the shortest pathway analysis of the GAT1587-mediated closed state of GIRK1/2. A total of 250 pathways are visualized. GIRK1-F97 serves as the source for the shortest pathway analysis. Important residues for function i.e., GIRK2 K194, K199, K200, and R230 serve as the sinks for the shortest pathway analysis. The thickness of the spline is weighted by the number of pathways that travel through that path.

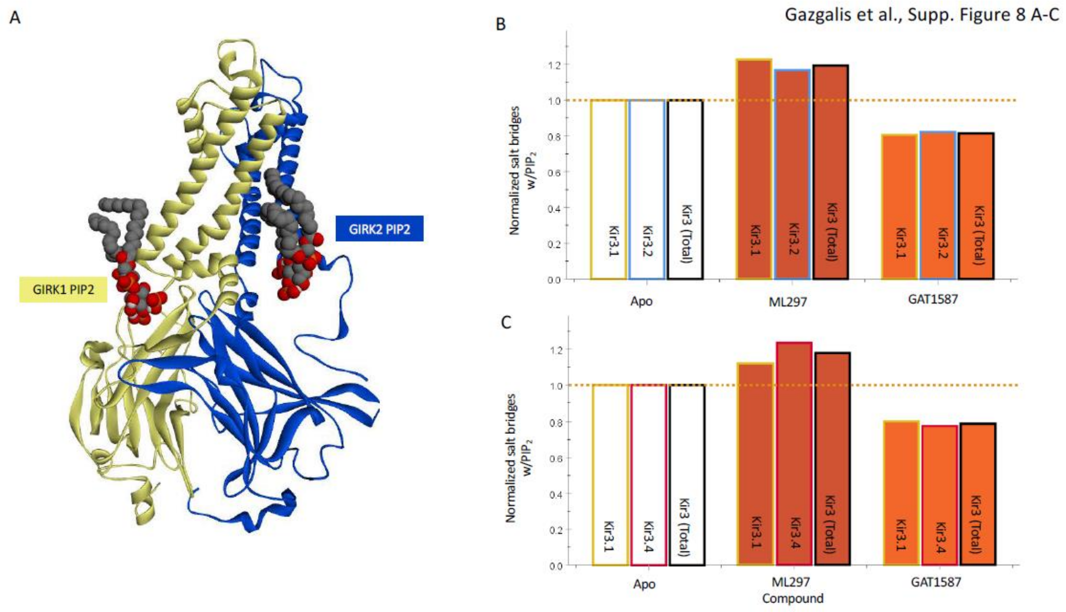

Figure S8. The ML297 versus GAT1587 effect on Kir3 channel-PIP$_2$ interactions is in opposite directions but to a similar extent. (**A**) PIP$_2$ interaction with the Kir3.1 or Kir3.2 (or Kir3.4 – not shown) subunit. (**B**) and (**C**) Effects of ML297 versus GAT1587 on normalized salt bridge formation between the channel and PIP$_2$ on a per subunit basis.

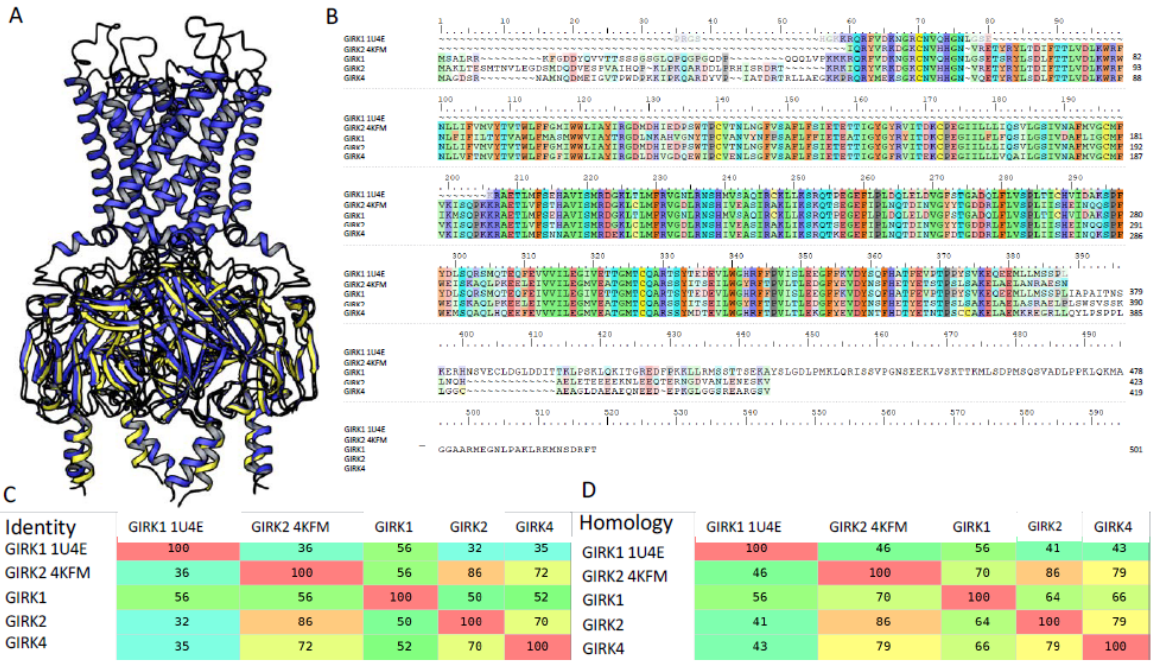

C

Identity	GIRK1 1U4E	GIRK2 4KFM	GIRK1	GIRK2	GIRK4
GIRK1 1U4E	100	36	56	32	35
GIRK2 4KFM	36	100	56	86	72
GIRK1	56	56	100	50	52
GIRK2	32	86	50	100	70
GIRK4	35	72	52	70	100

D

Homology	GIRK1 1U4E	GIRK2 4KFM	GIRK1	GIRK2	GIRK4
GIRK1 1U4E	100	46	56	41	43
GIRK2 4KFM	46	100	70	86	79
GIRK1	56	70	100	64	66
GIRK2	41	86	64	100	79
GIRK4	43	79	66	79	100

Figure S9. GIRK channel sequence and structural comparisons. (**A**) Structural alignment of high-resolution GIRK1 cytosolic domains (1U4E in yellow) and a terminal-end truncated GIRK2 (residues 52-380) that is normally 414 aa long (in blue). (**B**) Sequence alignment of GIRK1/2/4 highlighting by color different types of conserved amino acids (e.g., basic: in blue; acidic: in red; aromatic: in orange; etc.), including the sequences of the two high resolution structures shown in panel (**A**). (**C**, **D**) The five GIRK channels shown in panel (**B**) were analyzed for percent identity (**C**) and percent similarity (**D**).

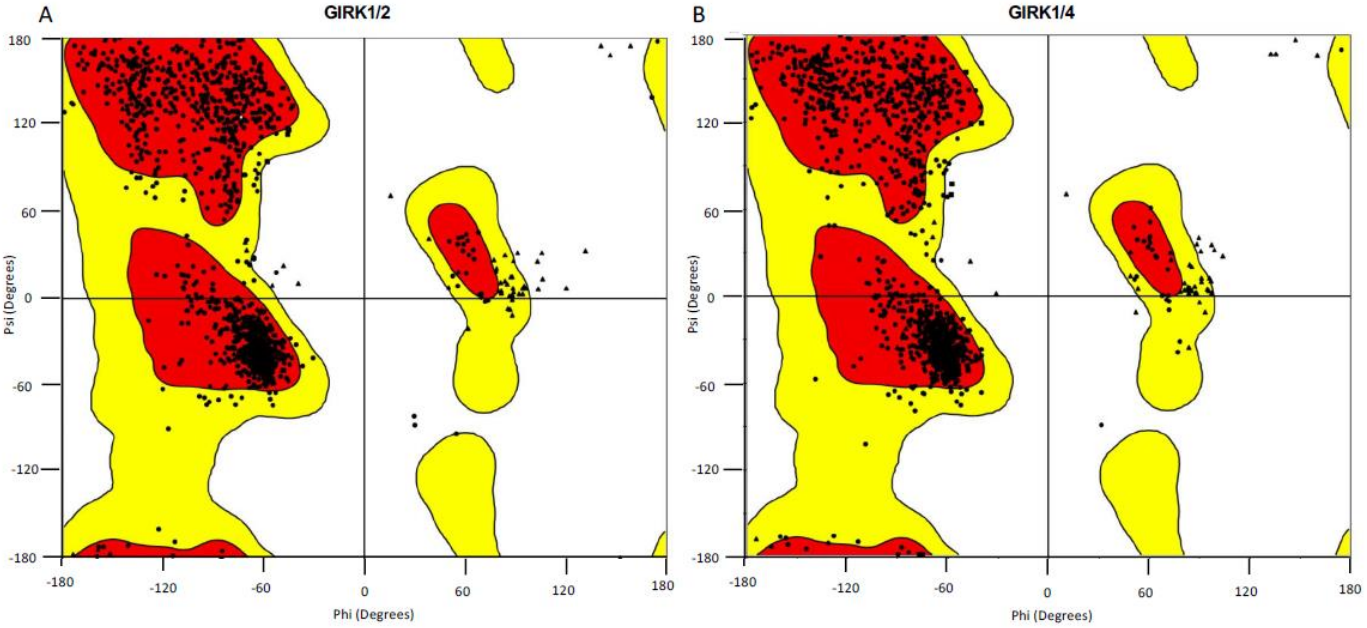

Figure S10. Ramachandran plots for generated homology models of the GIRK1/2 (**A**) and the GIRK1/4 (**B**) heterotetramers.

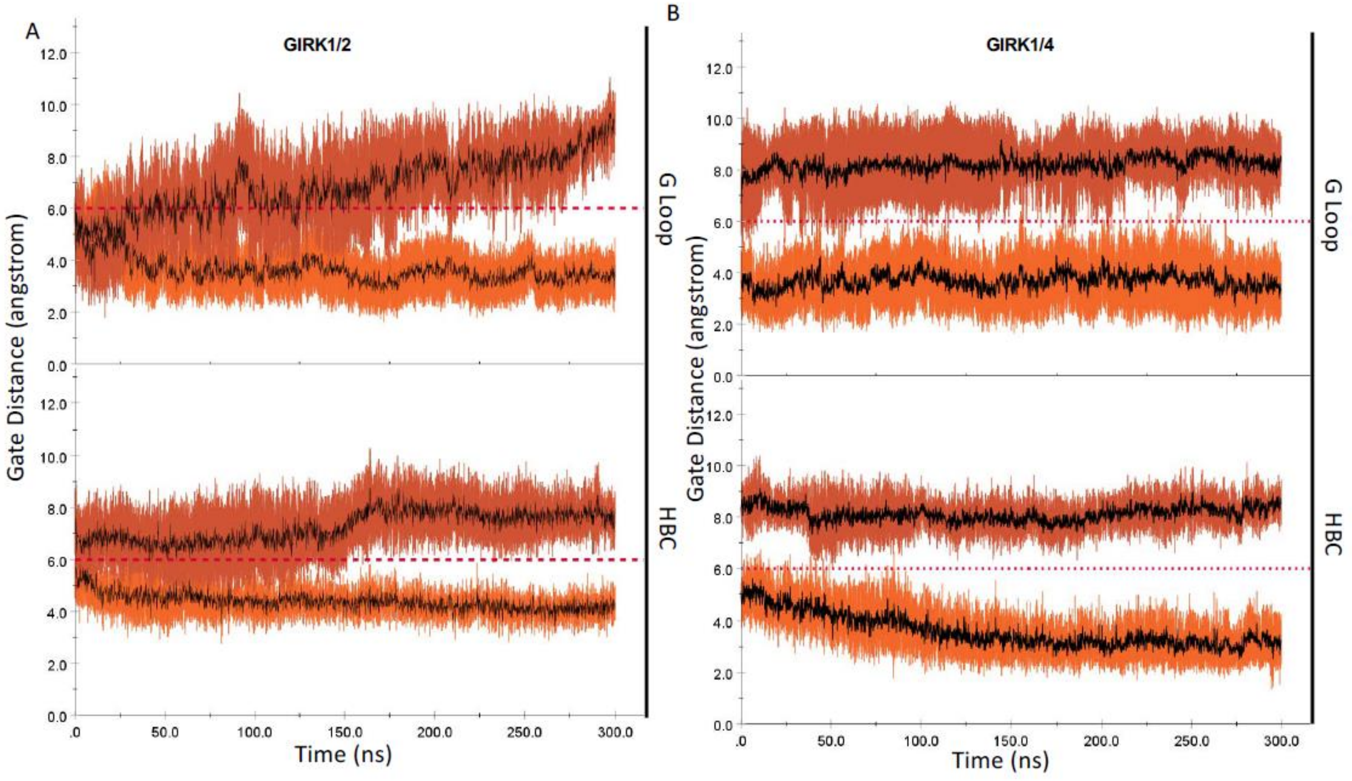

Figure S11. Gate distance statistics of the final 30 frames (~5 ns) from each of the 6-replica MD runs for GIRK1/2 and GIRK1/4 in the presence of either ML297 or GAT1587. (**A**) Pooled statistics from the 6 poses for the channel gate distances on the GIRK1/2. (**B**) Pooled statistics from the 6 poses for the channel gate distances on the GIRK1/4. The shaded region indicates the 95% confidence interval. The dark orange indicates ML297-activated systems. The light orange indicates GAT1587-inhibited systems.

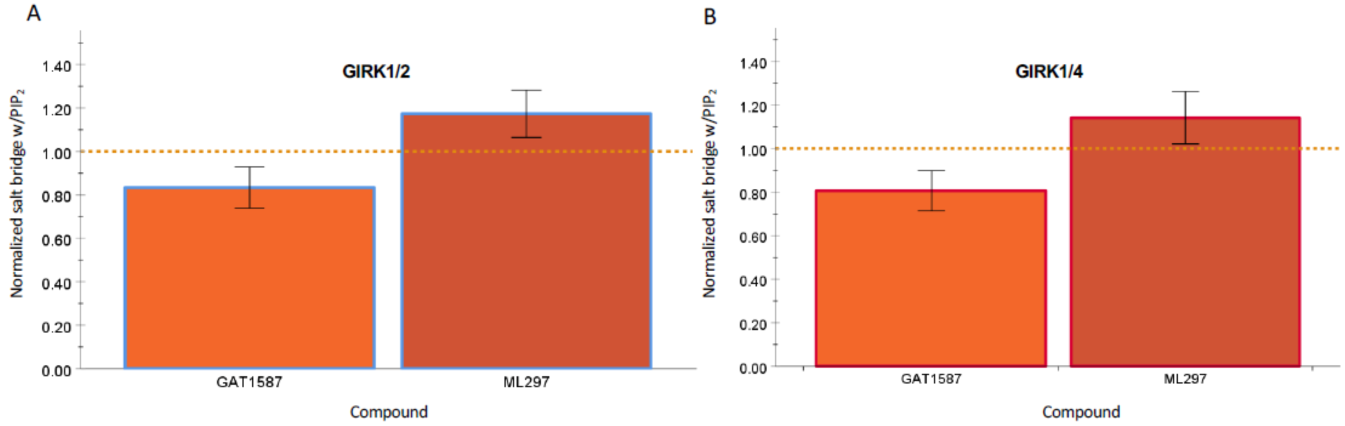

Figure S12. Channel-PIP2 interaction statistics of the final 30 frames (~5 ns) from each of the 6-replica MD runs for GIRK1/2 and GIRK1/4 in the presence of either ML297 or GAT1587. (**A**) Pooled statistics for the PIP2 normalized salt bridge formation over the course of the 6 simulations for GIRK1/2. (**B**) Pooled statistics for the PIP2 normalized salt bridge formation over the course of the 6 simulations for GIRK1/4. Salt bridge formation was normalized to the apo state reported in the main body of the text.

A

	Sum of Squares	df	Mean Square	F	Sig.
Between Groups	3118.378	1	3118.378	2717.876	.001
Within Groups	424.523	370	1.147		
Total	3542.901	371			

C

	Sum of Squares	df	Mean Square	F	Sig.
Between Groups	1073.822	1	1073.822	1939.242	.001
Within Groups	204.881	370	.554		
Total	1278.704	371			

B

	Sum of Squares	df	Mean Square	F	Sig.
Between Groups	2078.032	1	2078.032	2369.295	.001
Within Groups	324.515	370	.877		
Total	2402.547	371			

D

	Sum of Squares	df	Mean Square	F	Sig.
Between Groups	2681.724	1	2681.724	5993.291	.001
Within Groups	165.558	370	.447		
Total	2847.282	371			

E

	Sum of Squares	df	Mean Square	F	Sig.
Between Groups	1.383	1	1.383	23.683	.001
Within Groups	2.686	46	.058		
Total	4.068	47			

F

	Sum of Squares	df	Mean Square	F	Sig.
Between Groups	1.346	1	1.346	20.852	.001
Within Groups	2.970	46	.065		
Total	4.317	47			

Table S1. Statistical test for last 30 frames of each simulation. ANOVA was used to test for significance at the 0.05 level. The DF refers to the degrees of freedom of the dataset. F refers to the F statistic used in ANOVA statistical tests. Sig. refers to the significance value. A value of 0.001 indicates a significance P value of < 0.001. (**A**) G loop of the GIRK1/2 between the ML297 and GAT1587 simulations, (**B**) HBC of the GIRK1/2 between the ML297 and GAT1587 simulations,(**C**) G loop of the GIRK1/4 between the ML297 and GAT1587 simulations, (**D**) HBC of the GIRK1/4 between the ML297 and GAT1587 simulations, (**E**) Normalized salt bridge formation of the GIRK1/2 between the ML297 and GAT1587 simulations, (**F**) Normalized salt bridge formation of the GIRK1/4 between the ML297 and GAT1587 simulations
